# Whole genome sequences are required to fully resolve the linkage disequilibrium structure of human populations

**DOI:** 10.1186/s12864-015-1854-0

**Published:** 2015-09-03

**Authors:** Reuben J. Pengelly, William Tapper, Jane Gibson, Marcin Knut, Rick Tearle, Andrew Collins, Sarah Ennis

**Affiliations:** Human Genetics & Genomic Medicine, Faculty of Medicine, University of Southampton, Duthie Building (MP 808), Tremona Road, Southampton, SO16 6YD UK; Centre for Biological Sciences, Faculty of Natural & Environmental Sciences, University of Southampton, Southampton, UK; Complete Genomics, Inc., Mountain View, CA USA

**Keywords:** Linkage disequilibrium map, Population structure, Whole-genome sequencing, Recombination, Next-generation sequencing

## Abstract

**Background:**

An understanding of linkage disequilibrium (LD) structures in the human genome underpins much of medical genetics and provides a basis for disease gene mapping and investigating biological mechanisms such as recombination and selection. Whole genome sequencing (WGS) provides the opportunity to determine LD structures at maximal resolution.

**Results:**

We compare LD maps constructed from WGS data with LD maps produced from the array-based HapMap dataset, for representative European and African populations. WGS provides up to 5.7-fold greater SNP density than array-based data and achieves much greater resolution of LD structure, allowing for identification of up to 2.8-fold more regions of intense recombination. The absence of ascertainment bias in variant genotyping improves the population representativeness of the WGS maps, and highlights the extent of uncaptured variation using array genotyping methodologies. The complete capture of LD patterns using WGS allows for higher genome-wide association study (GWAS) power compared to array-based GWAS, with WGS also allowing for the analysis of rare variation. The impact of marker ascertainment issues in arrays has been greatest for Sub-Saharan African populations where larger sample sizes and substantially higher marker densities are required to fully resolve the LD structure.

**Conclusions:**

WGS provides the best possible resource for LD mapping due to the maximal marker density and lack of ascertainment bias. WGS LD maps provide a rich resource for medical and population genetics studies. The increasing availability of WGS data for large populations will allow for improved research utilising LD, such as GWAS and recombination biology studies.

**Electronic supplementary material:**

The online version of this article (doi:10.1186/s12864-015-1854-0) contains supplementary material, which is available to authorized users.

## Background

Detailed analysis of the linkage disequilibrium (LD) structure of human populations has been vital for the successful mapping of many human disease genes, understanding mechanisms underlying genetic recombination and elucidating patterns of selection and population structure [1]. The development of array-based genotyping (ABG) panels of single nucleotide polymorphisms (SNPs) enabled genome-wide association studies (GWAS) to localise numerous genetic variants with roles in human disease. Recognition that the genome contains ‘blocks’ of low haplotype diversity [2] facilitated the selection of ‘tagging’ SNPs [3] to enable cost-effective genotyping using panels of 500,000 to one million SNPs. Extensive SNP genotyping enabled the International HapMap Project to characterise the LD structure of diverse human populations [1]. The first LD maps of human chromosomes showed a haplotype block structure punctuated by ’steps’ aligning with recombination hotspots [4, 5]. The strong alignment of linkage and LD maps confirms historical recombination as the major determinant of LD structure [5–7].

Array-based LD maps of human chromosomes contain regions with negligible apparent LD between adjacent markers, seemingly reflecting high regional recombination, which are not well defined in the maps. Service et al. [7] assessed the impact of increasing marker density in a number of these regions using ABG data and found that some, though not all, regions were resolved with increasing marker density. For chromosome 22, 53 % of these regions were resolved using 27,060 *vs.* 9658 SNPs. Differences between populations were apparent, with LD maps from isolated populations (therefore having more extensive LD) containing substantially fewer such regions. Tapper et al. [6] constructed genome-wide LD maps using ~500,000 SNP genotypes from 60 HapMap samples with European ethnicity, identifying 3144 poorly resolved regions genome-wide and estimated that ~40,000 markers per Morgan would be needed to fully characterise LD structure. Assuming the autosomal linkage map length is ~33 Morgans [8] this suggests that ~1.3 million SNPs genome-wide would be sufficient to resolve these regions in this population. However, this assumes uniform marker spacing and LD intensity, whilst in reality much higher local marker density may be required for some of these regions. A particular difficulty exists for populations which have reduced LD due to extended population history, such as those from Sub-Saharan Africa, for which considerably higher marker coverage is required for complete coverage.

Given that whole-genome next generation sequencing (WGS) provides maximal genotype density, we consider the advantages of WGS-derived SNP genotypes for the characterisation of LD structure in different populations. We construct LD maps according to the Malécot-Morton model, using the program LDMAP [5, 6]. This model is defined as:

$$ \widehat{p}=\left(1-L\right)M{e}^{-\in d}+L $$

where $\widehat{p}$ is the association between SNPs, the asymptote *L* is the ‘background’ association between unlinked markers which is increased in small sample sizes and with residual population structure, *M* reflects association at zero distance with values ~1 consistent with monophyletic origin and <1 with polyphyletic inheritance, ϵ is the rate of LD decline, and *d* is the physical distance in kilobases between SNPs [5].

LDMAP constructs maps in linkage disequilibrium units (LDU, equal to ϵ*d*) such that one LDU corresponds to the (highly variable) physical distance over which LD declines to background levels. LDU plotted against the chromosome location forms step-like patterns with intense breakdown in LD, canonically due to recombination hotspots, and plateaus for broader regions of low haplotype diversity (blocks). Overall LDU map lengths are proportional to time since an effective population bottleneck [7, 9]. Hence, populations with shorter LDU maps have been founded more recently, experienced a more recent selective sweep, or have a smaller effective population size (such as some population isolates) compared to those with longer maps (such as Sub-Saharan African populations). The close correspondence between LD patterns and the linkage map reflects the dominant role of recombination in LD structure. In contrast to linkage maps, which are derived from family data and describe recombination over recent generations, LD maps are constructed from population data and reflect the historical impacts of recombination, mutation, selection and population history. Our findings show that WGS based LD maps provide greatly increased resolution of LD structure in both populations and indicate some genome regions in ABG-derived maps are incompletely covered. The findings have implications for interpretation in genome-wide association studies (GWAS) and support the use of WGS for association mapping and for establishing LD structure for studies of mechanisms underlying recombination and for identifying genomic regions subject to selection.

## Results

To investigate the impact of using WGS data for defining patterns of LD, we utilised publicly available WGS genotype data for chromosome 22 within the 1000 Genomes Project (henceforth referred to as the WGS dataset), and array-based genotype data from the International HapMap Project Phase 3 (henceforth the ABG dataset) [10, 11]. Due to its small size, chromosome 22 exhibits the highest recombination intensity in the genome [6] whereby LD declines sharply with distance and the LD maps are thus particularly sensitive for demonstrating the impact of the increased marker density in WGS data. We analysed LD maps constructed from CEU (Utah Residents (CEPH) with Northern and Western European ancestry) and YRI (Yoruba in Ibadan, Nigeria) populations. These are representative of populations which have developed since the effective ‘out of Africa’ bottleneck (CEU) and Sub-Saharan Africans (YRI). SNP markers within these datasets were filtered as described in Methods; final marker counts for each are given in Table 1. A detailed breakdown of marker attrition through filtering is presented in Additional file 1: Table S1.

**Table 1:** Number of individuals, component marker counts and LD map length and using ABG and WGS data

		Individuals	Markers	Map length (LDU)
ABG	CEU	112	15359	850.07
	YRI	147	16083	993.80
WGS	CEU	96	66704 (4.34)	1021.07 (1.20)
	YRI	80	91320 (5.68)	1569.46 (1.56)

Fold change *vs.* ABG data in parentheses

### LD map topography

**Fig. 1:**
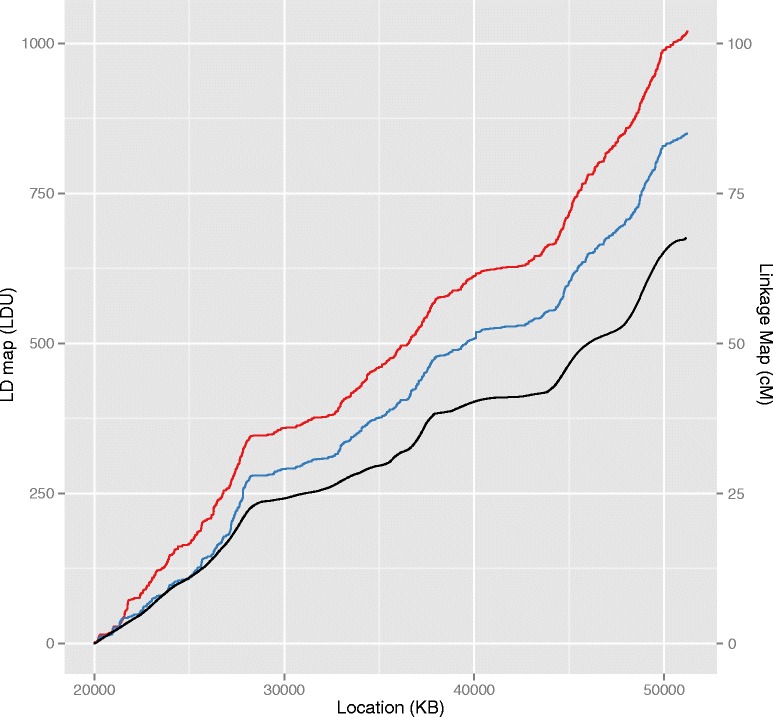
Comparison of LD maps from ABG and WGS, and linkage map. Comparison of WGS (red) and ABG (blue) CEU LD maps (left ordinate axis scale) and linkage map (black; right ordinate axis scale) for chromosome 22. Linkage map shown is from the June 2012 release of the Rutgers Map v3, interpolated using the Kosambi function (available at http://compgen.rutgers.edu/download_maps.shtml) [12]

LD maps produced using the ABG and WGS CEU datasets appear topographically highly similar when plotted, though with differing overall map lengths (Fig. 1). Regions of concordant strong LD are apparent, seen as low gradient regions in the plot, as well as regions of weak LD, appearing as a steep gradient. In addition, both maps appear to have similar contours to the linkage map produced from European samples, with broad areas reflecting strong and weak LD/recombination, [12]. It is noteworthy that there is an increased overall map length for the CEU WGS map compared to the ABG map (1.2 fold, Table 1). The change in map length is concurrent with much greater increases in marker density (4.3 fold) from ABG to WGS datasets.

**Fig. 2:**
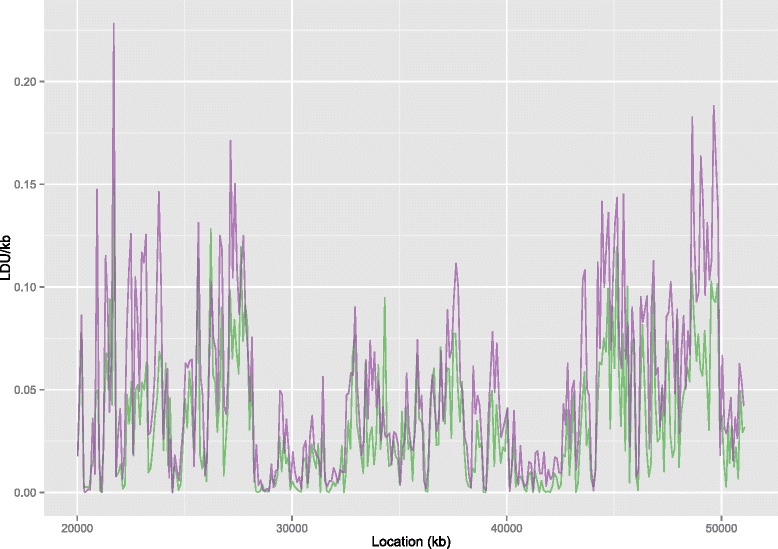
Comparison of LD decline intensity in WGS derived LD maps between populations. Comparison of regional rates of LD breakdown for CEU (green) and YRI (purple) populations using the WGS dataset for chromosome 22 for 100 kb windows. A very strong correlation between the LDU/kb for the two populations can be seen (*p* = 0.91, *p* < 2.2x10^−16^)

LD maps for the two WGS populations also show close alignment in LD structure with broad shared regions of stronger and weaker LD. When the LDU maps are represented as a rate (LDU/kb) in 100 kb windows (Fig. 2) the positions of the peaks, where LD declines rapidly, align closely between the two populations, as do regions with strong LD (low LDU/kb). The much longer LDU map for the YRI population reflects population history with increased time to erode LD through recombination, mutation and other processes [9]. There is a particularly marked increase in length for the YRI map of 1.6 fold from ABG to WGS data sets (Table 1).

### Marker density and frequency

**Fig. 3:**
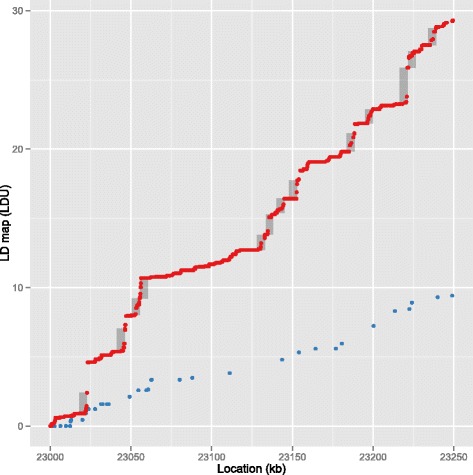
Expanded comparison of LD maps for a small region. Fine detail comparison of WGS (red) and ABG (blue) LD maps for a 250 kb region of YRI chromosome 22. All markers are plotted individually; hotspots are highlighted in grey. Whilst 13 hotspots are identified within the WGS map for this region, the ABG map shows no hotspots

The WGS data provides up to a 5.7 fold increase in number of markers compared to ABG data (Table 1; Additional file 1: Table S1). This increase in marker density allows greatly improved resolution of the LD maps in many regions. Although whole-chromosome LD map contours of ABG and WGS derived maps look very similar, noteworthy differences exist at higher resolution. Figure 3 shows an expanded view of a 250 kb region of the YRI population maps. The map of this region generated from the lower density ABG data failed to resolve 13 hotspots which are discernible in the WGS-based map. Many such narrow regions of high recombination can be far more accurately located using WGS maps.

**Fig. 4:**
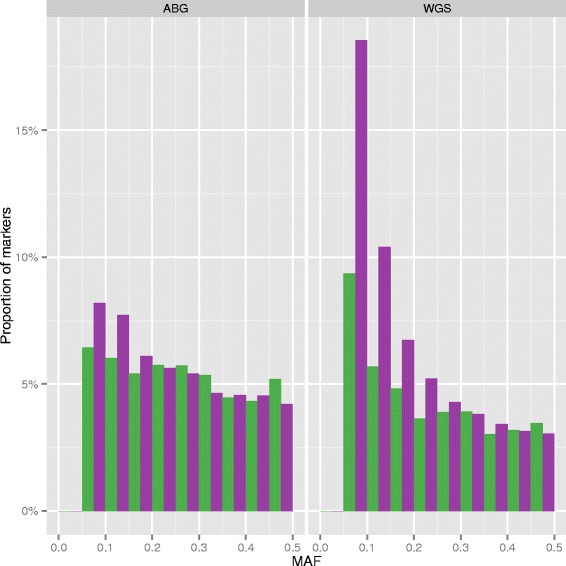
Distribution of allele frequencies between data sources. Histogram showing MAF distributions within ABG (left panel) and WGS (right panel) datasets for CEU (green) and YRI (purple) populations. A MAF bin width of 0.05 has been used. The median MAF for CEU is 0.25 and 0.21 for the ABG and WGS data respectively; the same metrics for the YRI are 0.23 and 0.15 respectively

As well as increased marker density in the WGS data, there is also a shift in the minor allele frequency (MAF) spectrum of the component markers (Fig. 4). The WGS dataset shows a significant reduction in the median MAF compared to the ABG data (*p* < 2.2 x10^−16^ for each population), with a far greater magnitude change in the YRI population compared to the CEU population (with a 35 and 18 % reduction in median MAF respectively). These data illustrate that: 1) markers at the lower frequency end of the range are particularly underrepresented in the arrays used to genotype the HapMap samples; and 2) this underrepresentation is most pronounced for the YRI population.

### Effect of population sample size

We investigated the extent to which population sample size within the WGS datasets impacts the marker density available for map generation, as well as the length of the final LD maps. For 12 Mb of the chromosome we generated random subsets of the full datasets with varying sample size, and then performed marker filtering and map generation as described. With an increased sample size, a higher marker density is achieved for map generation, with diminishing returns with larger sample sizes (Additional file 1: Figure S1). From these data, we extrapolated the sample size for which the addition of 10 individuals increases marker density by <1 %; this marker saturation is achieved with 90 and 110 individuals for the CEU and YRI populations respectively.

For maps from these data subsets, there is a weak, but significant, correlation between sample size and LDU length of the resultant CEU maps (Additional file 1: Figure S2); the YRI maps show no significant correlation. This indicates that overall map lengths are largely robust to variations in sample size. Due to the increased marker diversity of the YRI cohort compared to the CEU, a greater number of individuals need to be sampled for complete marker saturation. At smaller sample sizes however, the deviation of map lengths from average is much broader, reflecting increased sensitivity to heterogeneity within the dataset (Additional file 1: Figure S3). Despite the increased map variability, the WGS map remains consistently longer than the corresponding ABG map. Even where maximal marker densities have been attained, larger sample sizes are likely to improve the population representativeness of the map.

### Fine map structure comparison between ABG and WGS

To compare LD structure between ABG and WGS maps we segmented the LD maps into non-overlapping 100 kb regions (Additional file 1: Table S2). All LD maps show a very strong correlation with all other maps (ρ > 0.87), with stronger correlations within population.

In all cases, the correlation with the linkage map is also strong (ρ = 0.56–0.60); this correlation is likely lower due to the lower resolution of the linkage map and components of the LD structure that are not due to recombination. We find a particularly strong correlation (*p* = 0.94, *p* < 2.2x10^−16^) in the lengths of these segments in LDUs between the two YRI data sources. The increase in LD map length for the WGS YRI map might be partly attributed to the greatly increased marker density, however there is only a relatively weak, though strongly significant, correlation between increase in marker density and increase in LDU length in these 100 kb regions (*r*^2^ = 0.19, *p* < 2.2x10^−16^; Additional file 1: Figure S3). A total of 37.5 % of 100 kb regions show negligible change in LDU length (< |1|) despite greatly increased marker density, suggesting a large proportion of the chromosome is approaching complete marker saturation in the ABG data. However, other regions show substantially increased LDU length (with many regions increased by over 5 LDU) with the higher marker density, suggesting they are poorly resolved in array-based maps.

The 100 kb regions in the YRI data which exhibit the largest and smallest magnitude LDU length change (10 of each) between ABG and WGS maps were further investigated (Additional file 1: Figure S4). Regions with large LDU increase in the WGS data contain SNPs with a significantly higher MAF than regions with a small change (*p* = 5.7x10^−7^, median of 0.18 and 0.13 for the large and small magnitude change regions respectively), no significant difference between the MAF distributions of these regions was observed in the ABG data (*p* = 0.39). This indicates that while there is particular enrichment of lower frequency markers using the WGS data, it is the inclusion of common variation absent from array panels which has the largest effect on the resulting LD map. The exclusion of highly LD informative common variation in array-based panels may reflect the ascertainment of tagging SNPs which is not optimised for all populations.

### Hotspot identification

The LD landscape is known to comprise long regions of low haplotype diversity punctuated by very narrow regions of LD breakdown which align with recombination hotspots. WGS-based maps allow for more complete resolution of recombination hotspots compared to ABG-based maps (Fig. 3). We therefore systematically evaluated hotspots identified in the four LDU maps. We defined hotspots as five kb regions containing SNPs which were separated by at least 1 LDU. In both populations, the WGS derived maps delimit a substantially increased number of hotspots (Additional file 1: Table S2). The CEU maps show a 1.7 fold increase in resolved hotspots, compared to 2.8 fold increase in the YRI maps. This indicates that array-based genotyping only partially resolves the LD structure in both populations and resolution is particularly incomplete for the YRI population.

**Fig. 5:**
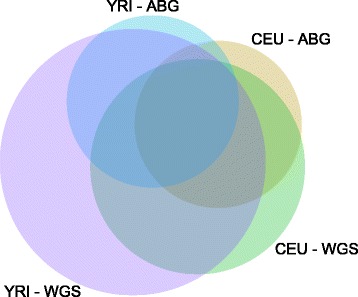
Concordance between identified hotspots. Euler diagram showing overlap between hotspots identified in each dataset. The area of all regions is proportional to the number of hotspots which are present in those sets; total area represents 629 independent hotspots across all datasets

We also assessed concordance between hotspots identified in the datasets (Fig. 5; Additional file 1: Table S2). The majority of hotspots identified in ABG data were also identified in the corresponding WGS maps (81 and 86 % for CEU and YRI maps respectively). However, for YRI only 38 % of hotspots identified in the WGS map were also represented in the corresponding ABG map. Furthermore, only 13 % of identified hotspots showed concordance across the four datasets, with 29 % of all hotspots only observed in the YRI WGS map. Of the 170 CEU hotspots identified in the ABG map the YRI ABG map identifies only 50 % while, in contrast, the YRI WGS map detects 70 %. This indicates that relatively poor resolution of the LD structure in the YRI array-based map suggests misleadingly low concordance between hotspot locations across the two populations. Leveraging WGS data will therefore enable more effective characterisation of LD structure for YRI, and other populations with an extended population history, for disease gene mapping and the functional analysis of genomes.

## Discussion

We have shown that WGS-derived data enables superior resolution of LD structure in two populations with distinct histories. The increased marker density provides much improved delineation of regions of high and low recombination. Although some chromosome regions are well represented in array-based maps, population specific increases in map lengths of ~20–60 % reflect improved WGS resolution of the LD structure in other regions. These seem likely to include regions highlighted as poorly characterised in earlier array-based maps [6, 7]. Similarly, Lau et al. [13] observed a ~3 % increase in map length when comparing maps generated from HapMap phases 1 and 2, with the associated increase in marker density.

We have shown that the YRI maps are improved by the greatest margin due to the inclusion of common variation excluded from the array-based genotyping panel. Array genotyping necessarily has a data acquisition bias; variants must be identified prior to array design, limiting the array capture to known variation which may be optimally informative for only the populations used for variant discovery. This ascertainment bias can cause issues in population genetic studies particularly where array data of a population not included in variation discovery is being investigated [14, 15]. Recently developed arrays which include data from the three HapMap phases, along with variants identified in the 1000 Genomes Project, achieve coverage of common variation of 92–93 % for CEU but only 76 % for YRI [16].

The evidence presented here indicates that the YRI LD structure is particularly poorly represented using array-based data, reflecting these unresolved biases in marker selection. While improvements in representativeness have been made, achieving good representation of all populations using ABG methodologies is intrinsically impracticable given technological and cost limitations on genotyping density. In contrast, using WGS there is negligible acquisition bias for variant discovery, though there can be bias where a population is highly divergent from the reference genome assembly; improvements in assembly and analytical tools should hopefully further reduce this bias in the near future [17]. Some regions are still however refractory to WGS analysis, such as repetitive regions, again, advances will continue to reduce these issues [18].

The total LD map length is relatively independent of number of samples. This indicates that although an increase in the number of homogenous individuals used in map generation improves accuracy, resolution and population representativeness, the underlying LDMAP algorithm provides robust maps with even small population samples as previously noted [19, 20]. This may prove invaluable where the ascertainment of large data samples is impractical.

The high diversity of African populations, which reflects a much longer effective population bottleneck time, offers a rich resource for analysis of LD structure. Increased historical recombination makes sub-Saharan African populations ideal for GWAS studies, particularly for post-GWAS refinement, as well as for basic research into recombination biology and selection. Poor representation of African LD structure is considered likely to impact reproducibility of GWAS results. Marigorta and Navarro [21] investigated GWAS-derived disease variant reproducibility across 28 diseases. While most loci and SNPs discovered in Europeans have been extensively replicated in European and East Asian populations, replication in African populations is much less frequent. At least a proportion of these failed replications reflect heterogeneity in LD between causal variants and the tag SNPs used in GWAS panels so selection of alternative tags specific to the population used may improve reproducibility.

The incomplete resolution of LD structure in array-based LD maps which is evident even for the CEU population may have impacted the detection of disease variation in genome-wide association studies. With decreasing sequencing costs, WGS-based GWAS are becoming viable, with some successes reported [22]. These studies have the advantages of avoiding the marker ascertainment bias, and enable rare and common variation to be interrogated contemporaneously. Such studies may improve GWAS reproducibility, as well as identification of additional disease variation underlying some of the ‘missing heritability’ [23].

LD maps have been used successfully in GWAS for refinement of candidate regions [24, 25]. Sabatti et al. [25] defined regions of interest around nine newly identified disease genes underlying metabolic traits using a liberal four LDU window. Improvements in LD map resolution through the use of WGS data will substantially reduce the size of regions for targeted follow-up. To investigate the potential gains of using WGS-derived LD maps for fine mapping, we assessed the physical window size corresponding to four LDU for 172 GWAS association signals identified in European populations on chromosome 22 [26]. We considered the physical distance between the two nearest markers up and downstream which are at least two LDU away from the GWAS signal SNP. For the CEU population map WGS-based four LDU windows were, on average, 17 % smaller compared to the ABG map (262 *vs.* 316 kb respectively). Furthermore, if we presume these GWAS signals are reproducible in Sub-Saharan African populations, the average four LDU window is just 152 kb in the WGS YRI map, a further 42 % reduction in candidate region size compared to the CEU WGS map.

Considerably greater resolution can be achieved in fine-mapping using a population with African ancestry by exploiting the weaker LD as has been recently demonstrated in African American populations [27]. African populations have been historically underrepresented in population genetic studies but the African Genome Variation Project [28] is focussed on using whole-genome sequencing and other methods to refine the detection of disease variation in these populations. Construction of fully saturated whole genome LD maps from diverse African samples will undoubtedly improve efforts to map disease variants and help distinguish true population differences in genetic disease variation from those which have failed to replicate due to incomplete marker coverage in African samples.

## Conclusions

We have herein discussed several improvements to LD mapping attained using WGS data. Firstly, WGS data allows complete resolution of LD structure, given the maximal marker density. Secondly, as there is no ascertainment bias in genotypes, the data are also far more representative of the population under study, particularly notable for Sub-Saharan African populations. Thirdly, data from a larger number of individuals is required to best interrogate LD patterns in diverse populations, particularly those with long population history. We have shown that array-based SNP panels incompletely represent the LD structure in both populations studied and this may have impacted the success of genome-wide association studies for detecting disease variation. Genome-wide association studies using whole genome sequences may offer a route to capturing some of this additional variation.

## Methods

Publicly available 1000 Genomes Project [10] data derived from the *Complete Genomics* high depth whole-genome sequencing platform was used for WGS map generation [29]. WGS data for two population cohorts were used, namely the Utah Residents (CEPH) with Northern and Western European ancestry (CEU; 96 individuals), and Yoruba in Ibidan, Nigeria (YRI; 80 individuals). For comparison, array-derived HapMap Phase 3 release 3 data were also used [11]. ABG cohorts used were CEU (112 individuals), and YRI (147 individuals) samples. All individuals utilised for map generation were founders, and physical positions were defined according to GRCh37 (hg19) coordinates.

We consider here the region Chr22:20,000,000–51,304,566. The centromeric heterochromatin was excluded as these regions show very low density of polymorphic makers and complete LD, as well as a tendency for erroneous genotyping due to the repetitive nature of the sequences. Genotype data were filtered prior to map generation using PLINK [30] or VCFtools [31] to remove non-biallelic SNPs, SNPs with MAF within the dataset < 0.05, SNPs with Hardy-Weinberg equilibrium deviation *p*-value < 0.001 [32] and SNPs with > 5 % missing data. All statistical analyses were performed using R [33].

LD map generation was performed using the LDMAP program, with default parameters [20, 34]. For sample size reproducibility investigations, random subsets of the full cohort were generated and LD maps generated from the resulting dataset for three regions (Chr22:20,000,000–25,000,000, Chr22:30,000,000–35,000,000 and Chr22:45,000,000–47,000,000; 12 Mb total size) with 20 pseudoreplicates generated for each region. We restricted these analyses to 12 Mb of the chromosome due to the computational intensity of LD map generation. Following subsampling, filtering and LD map generation with a range of sample sizes, a negative exponential cumulative model was fitted to the marker density data for each population and extrapolated to estimate sample sizes required for effective map saturation. We defined map saturation as the sample size at which an additional 10 individuals provides less than 1 % increase in marker density.

We investigated regions of intense LD decline, which are canonically the product of high levels of historical recombination. Recombination hotspots are known to span just 1–2 kb [35, 36]. For comparison of LDU maps we defined a hotspot as a region of maximum size 5 kb in which there was at least a one LDU change between two encompassed SNPs, as observed in previous studies [37]. Hotspots were deemed concordant between datasets if there was any physical overlap; these liberal definitions were required due to the differing marker composition and density of datasets.

## Additional file

Additional file 1:
**Additional material as referenced in the text.** (PDF 257 kb)

## Abbreviations

ABG: Array-based genotyping
CEU: Utah Residents (CEPH) with Northern and Western European ancestry
GWAS: Genome-wide association study
LD: Linkage disequilibrium
LDU: Linkage disequilibrium unit
MAF: Minor-allele frequency
SNP: Single nucleotide polymorphism
WGS: Whole-genome sequencing
YRI: Yoruba in Ibadan, Nigeria
