# Supplementary material for: Whole genome sequences are required to fully resolve the linkage disequilibrium structure of human populations
Source: BMC Genomics. 2015 Sep 3;16(1):666. doi: 10.1186/s12864-015-1854-0 (PMC4558963; doi:10.1186/s12864-015-1854-0)
Supplement: Additional file 1: — Additional material as referenced in the text. (PDF 257 kb) [file 12864_2015_1854_MOESM1_ESM.pdf]

Supplementary Table S1 | Marker counts throughout filtering for all datasets

	ABG				WGS			
	CEU		YRI		CEU		YRI	
	**Count**	**Fold change**[d]	**Count**	**Fold change**	**Count**	**Fold change**	**Count**	**Fold change**
**Raw count**	17938	-	18906	-	214399	-	279848	-
**MAF**[a]	15420	0.86	16142	0.85	74946	0.35	106910	0.38
**HWE**[b]	17923	1.00	18887	1.00	211048	0.98	275780	0.99
**Missingness**[c]	17872	1.00	18906	1.00	198911	0.93	258517	0.92
**Final count**	15359	0.86	16083	0.85	66704	0.31	91320	0.33

[a]Markers with minor allele frequency < 0.05 within the cohort excluded.
[b]Markers with a Hardy-Weinberg equilibrium deviation p-value < 0.001 within the cohort.
[c]Markers with > 5% data missing excluded.
[d]Fold change is in comparison to the raw count for each filtering criterion in isolation.

Supplementary Table S2 | Spearman's rank correlations between LDU map lengths of 100 kb segments.

CEU-ABG	CEU-WGS	YRI-ABG	YRI-WGS	Linkage	
1	0.92	0.88	0.87	0.56	**CEU-ABG**
	1	0.89	0.91	0.58	**CEU-WGS**
		1	0.94	0.60	**YRI-ABG**
			1	0.59	**YRI-WGS**
				1	**Linkage**

$p < 2.2x10^{-16}$ for each correlation .

Supplementary Table S3 | Counts of hotspots in each dataset with corresponding hotspots identified in all other datasets

		ABG		WGS	
		**CEU**	**YRI**	**CEU**	**YRI**
**ABG**	**CEU**	**170**	86 (0.51)	137 (0.81)	119 (0.70)
	**YRI**	88 (0.50)	**176**	115 (0.65)	152 (0.86)
**WGS**	**CEU**	157 (0.53)	126 (0.43)	**296**	224 (0.76)
	**YRI**	149 (0.30)	187 (0.38)	244 (0.50)	**491**

Values shown indicate the number of hotspots in the dataset indicated with the row label with a corresponding hotspot(s) in the dataset indicated with the column label. Proportion of total hotspots recapitulated is shown in parentheses.

Supplementary Figure S1
Relationship between sample size and marker density.

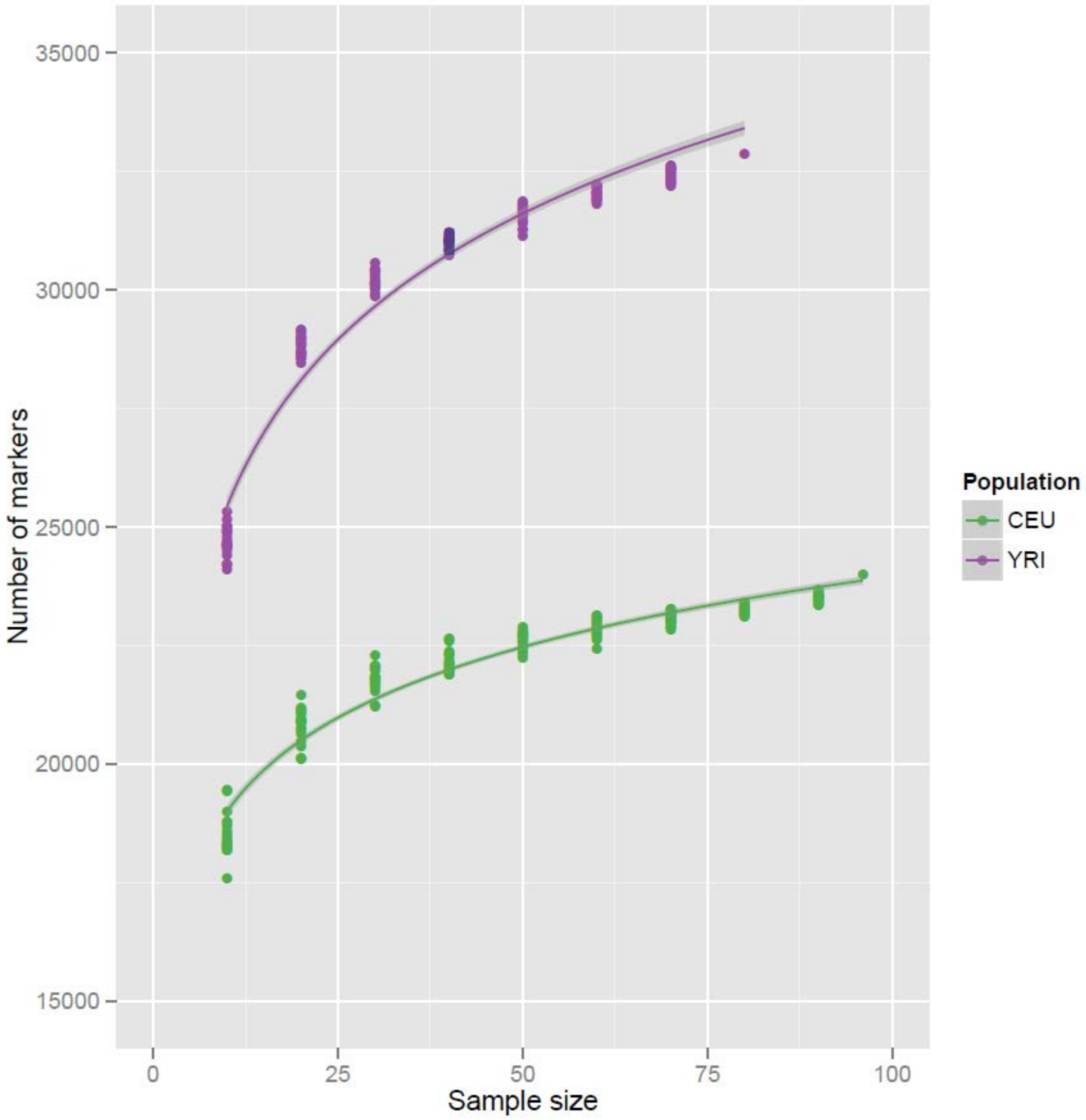

Supplementary Figure S1 | Relationship between sample size and marker density. Correlation between number of individuals sampled and number of markers for a 12 Mb region, in the WGS data for CEU and YRI populations. A negative cumulative exponential regression has been fitted ($r^2 > 0.94$, $p < 2.2x10^{-16}$ in both populations; shaded region indicates 95% confidence interval).

Supplementary Figure S2
Relationship between sample size and LD map length.

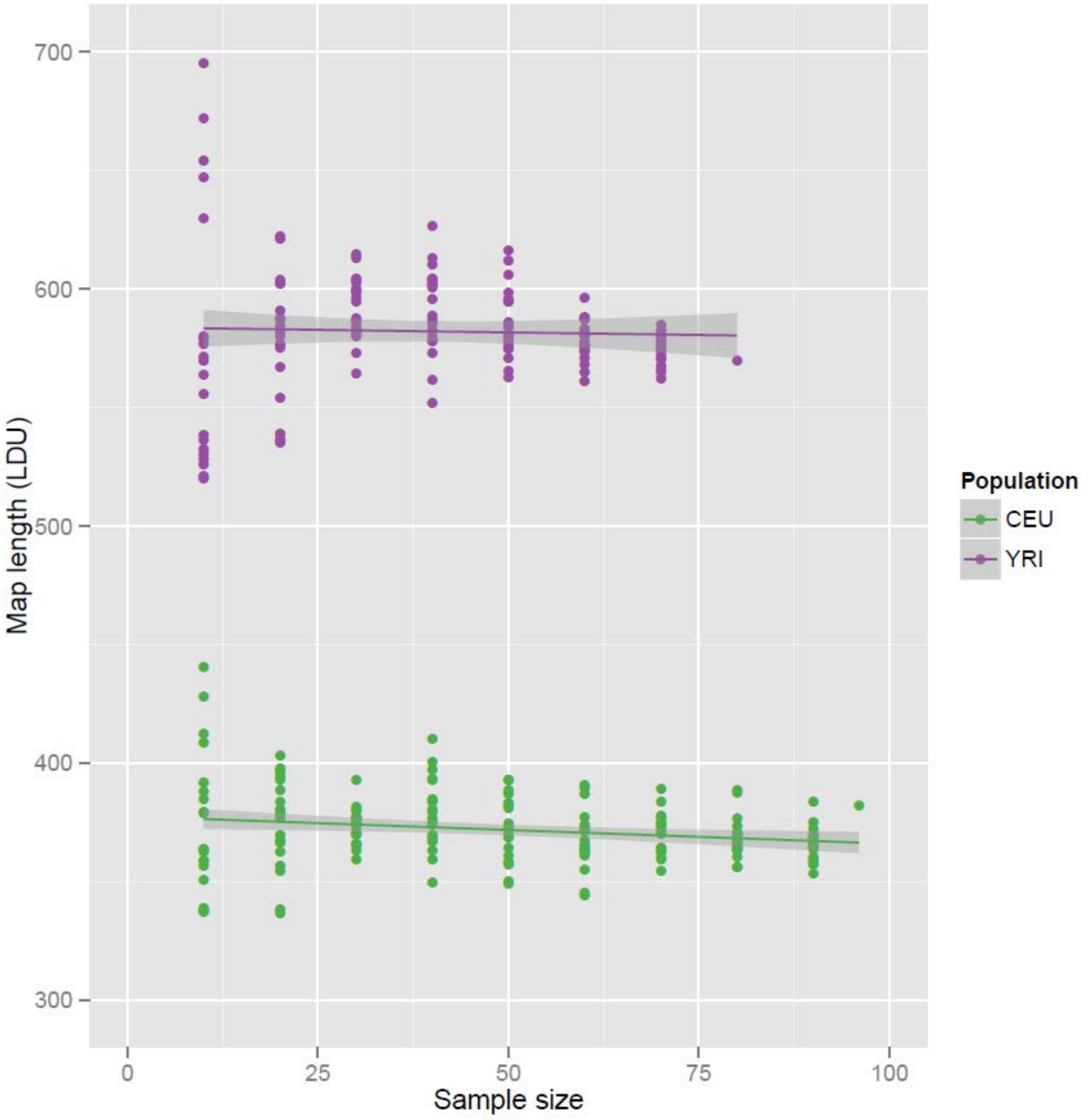

Supplementary Figure S2 | Relationship between sample size and LD map length. Correlation between number of individuals sampled and LDU map length for a 12 Mb region in the WGS data for CEU and YRI populations. A linear regression has been fitted ($r^2 = 0.04$, $p = 0.0087$ for CEU, gradient is not significantly different from zero for YRI ($p = 0.69$); shaded region indicates 95% confidence interval).

Supplementary Figure S3
Jackknife assessment of WGS datasets for varying sample sizes.

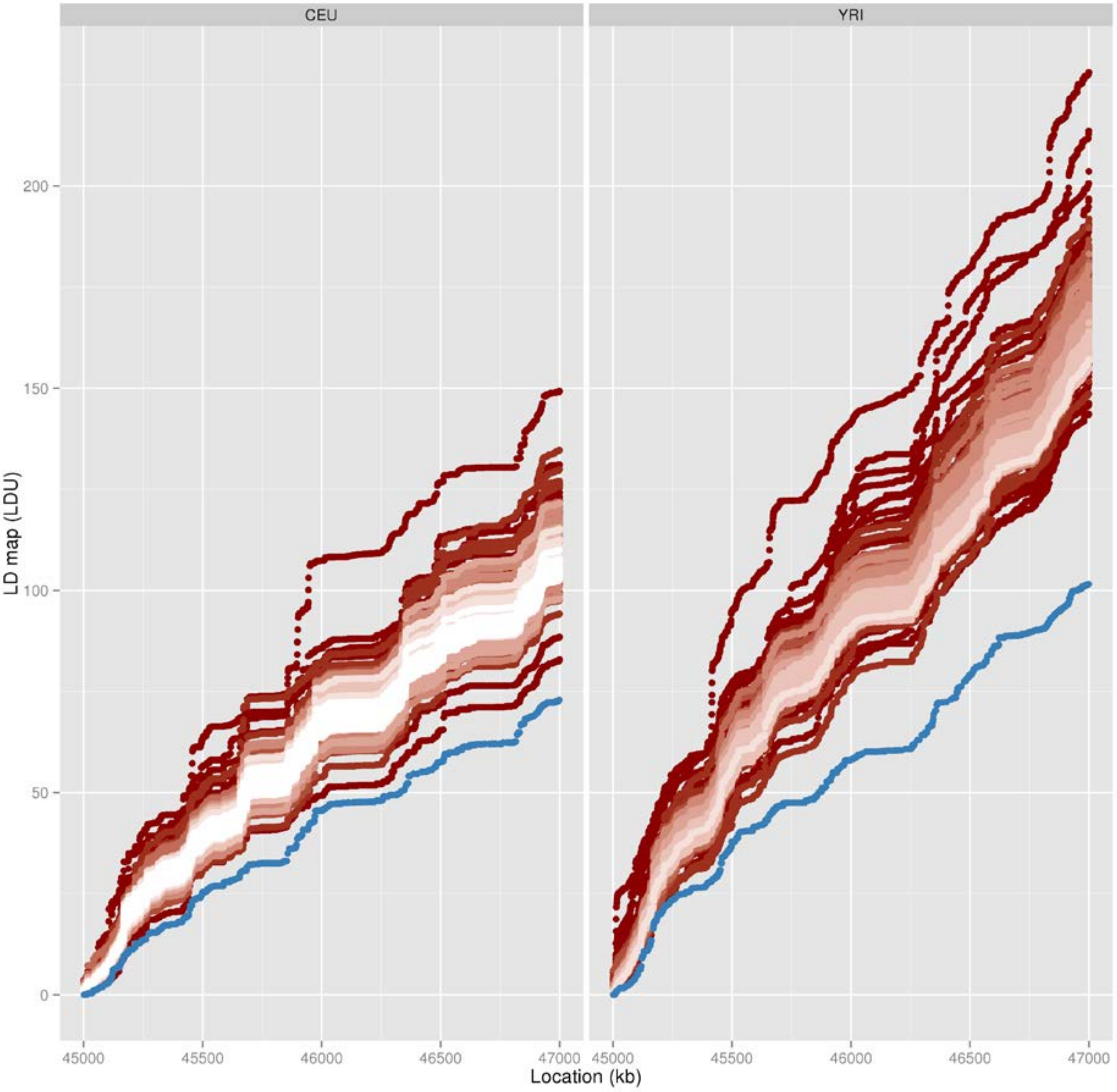

Supplementary Figure S3 | Jackknife assessment of WGS datasets for varying sample sizes. LD maps for a 2 Mb region constructed following random subsampling of WGS data for varying sample sizes (red to white with increasing sample size, range 10 to 90, with increments of 10 individuals) of both populations. For comparison, the ABG map is included (blue). Increasing variability in the WGS map can be seen in lower sample sizes, with the maps converging at larger sample sizes. Despite the increased variability at the smallest sample size of 10, the ABG map remains consistently shorter.

Supplementary Figure S4

Relationship between increase in marker density and increase in LD map length.

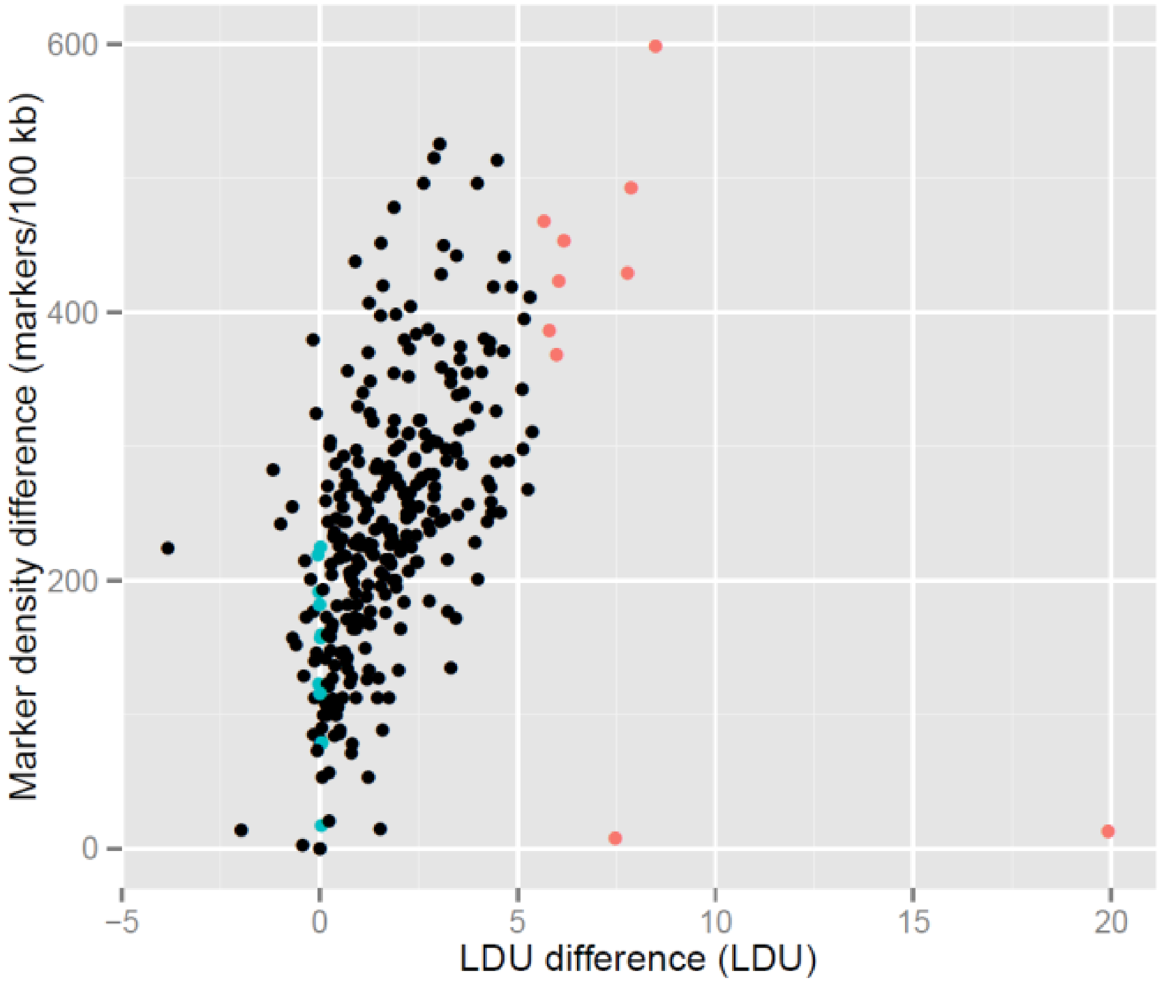

Supplementary Figure S4 | Relationship between difference in marker density difference in LD map length. Scatter plot showing change in LDU *vs.* change in marker density for 100 kb regions between ABG and WGS map from YRI datasets. The 20 regions selected for further analysis as regions of largest magnitude change (red) and those with minimal length change (blue) are shown. Note that two of the selected regions span 23,000 - 23,200 kb, shown in Figure 3. A total of 312 regions were assessed in total.
